# Neuroprotective Effects of Metformin Through the Modulation of Neuroinflammation and Oxidative Stress

**DOI:** 10.3390/cells14141064

**Published:** 2025-07-11

**Authors:** Sarah Reed, Equar Taka, Selina Darling-Reed, Karam F. A. Soliman

**Affiliations:** Division of Pharmaceutical Sciences, College of Pharmacy and Pharmaceutical Sciences, Institute of Public Health, Florida A&M University, Tallahassee, FL 32307, USA; sarah1.reed@famu.edu (S.R.); equar.taka@famu.edu (E.T.); selina.darling@famu.edu (S.D.-R.)

**Keywords:** neurodegeneration, neuroinflammation, oxidative stress, metformin, type 2 diabetes

## Abstract

Epidemiological studies have shown that individuals with type 2 diabetes have an increased risk of developing neurodegenerative diseases. These diseases and type 2 diabetes share several risk factors. Meanwhile, the antidiabetic drug metformin offers promising neuroprotective effects by reducing oxidative stress and neuroinflammation, two significant factors in neurodegenerative diseases. This review examines the mechanisms by which metformin mitigates neuronal damage. Metformin reduces neuroinflammation by inhibiting microglial activation and suppressing proinflammatory cytokines. It also triggers the nuclear factor erythroid-2-related factor-2 (Nrf2) pathway to combat oxidative stress, an essential regulator of antioxidant defenses. These outcomes support the possible neuroprotective roles of metformin in type 2 diabetes-related cognitive decline and conditions like Alzheimer’s disease. Metformin’s therapeutic potential is further supported by its capacity to strengthen the blood–brain barrier’s (BBB’s) integrity and increase autophagic flux. Metformin also offers several neuroprotective effects by targeting multiple pathological pathways. Moreover, metformin is being studied for its potential benefits beyond glycemic control, particularly in the areas of cognition, Alzheimer’s disease, aging, and stroke management. Evidence from both clinical and preclinical studies indicates a complex and multifaceted impact, with benefits varying among populations and depending on underlying disease conditions, making it an appealing candidate for managing several neurodegenerative diseases.

## 1. Introduction

Numerous neurodegenerative diseases, including Alzheimer’s disease, are frequently linked to oxidative stress and chronic neuroinflammation. Neurodegenerative diseases cause damage to the nerve cells of the central and peripheral nervous systems (CNS and PNS), leading to degeneration and a gradual loss of their ability to function [1]. Oxidative stress is a result of free radical overproduction. It is caused by an inability to produce sufficient antioxidants. Microglia, the immune cells responsible for regulating neuronal networks, can repair injuries and regulate development in the brain. They are typically activated in response to neuroinflammation [2].

Metformin, which is frequently administered to treat type 2 diabetes, has garnered increased attention lately because of its possible neuroprotective benefits. According to several studies, metformin may modulate neuroinflammation and oxidative stress and activate the nuclear factor erythroid-2-related factor-2 (Nrf2), a transcription factor implicated in cellular defense mechanisms [3]. Understanding how metformin interacts with these pathways is necessary to clarify its therapeutic potential in neurodegenerative diseases.

Neuroinflammation is one of the leading causes of neuronal damage in several neurodegenerative diseases, such as Alzheimer’s disease. Chronic neuroinflammation is characterized by the excessive activation of microglia, which causes a release of proinflammatory cytokines, subsequently contributing to neuronal damage and loss [4]. Much interest has been in metformin’s capacity to control the inflammatory response. One unique way metformin can be used in neuroprotection is its ability to cross the blood–brain barrier. Several studies have suggested that metformin can modulate various molecular pathways, including the activation of adenosine-monophosphate-activated protein kinase (AMPK), which is essential for inflammation and cellular energy regulation. When AMPK is activated, it inhibits the NF-κB signaling pathway, decreasing the generation of inflammatory cytokines and promoting a neuroprotective environment [5]. Additionally, metformin improved spatial memory and maintained neuronal integrity by significantly reducing neuroinflammation, reactive gliosis, and hippocampal neuron loss [6]. Recent studies have shown that metformin ameliorates the neuroinflammatory environment for neurons and astrocytes by suppressing the release of proinflammatory mediators, like tumor necrosis factor α (TNFα) and interleukin-1β (IL-1β), and upregulating anti-inflammatory mediators, like interleukin-10 (IL-10) and interleukin-4 (IL-4) [3].

Oxidative stress is another major cause of many neurodegenerative diseases. It is caused by the body’s inability to detoxify a harmful load of reactive oxygen species (ROSs). The excessive load of oxidative stress has been associated with the development of neurodegenerative diseases and cognitive impairment [7]. Metformin has demonstrated the ability to counteract oxidative stress through several mechanisms, including activating the AMP-activated protein kinase (AMPK) signaling pathway and Nrf2 activation, boosting antioxidant defenses [8]. Therefore, by increasing the expression of antioxidant proteins and enzymes, Metformin can reduce oxidative damage to brain cells, suggesting that it may combat oxidative stress.

The Nrf2 transcription factor controls the body’s defenses against inflammation and oxidative stress [9]. Normally, Nrf2 is sequestered in the cytoplasm and targeted for degradation; however, when exposed to oxidative stress or electrophilic substances, Nrf2 translocates to the nucleus, where it stimulates the production of genes encoding for antioxidant proteins, detoxifying enzymes, antiapoptotic proteins, and proteasome proteins [10,11]. Many of the cellular protective effects of metformin are attained by modifying Nrf2 [12]. Therefore, these research findings suggest that metformin’s neuroprotective properties may be beneficial in managing conditions like Alzheimer’s disease, Parkinson’s disease, and other neurodegenerative disorders where oxidative stress and inflammation are prominent.

## 2. Metformin and Its Therapeutic Application in Type 2 Diabetes

**Figure 1:**
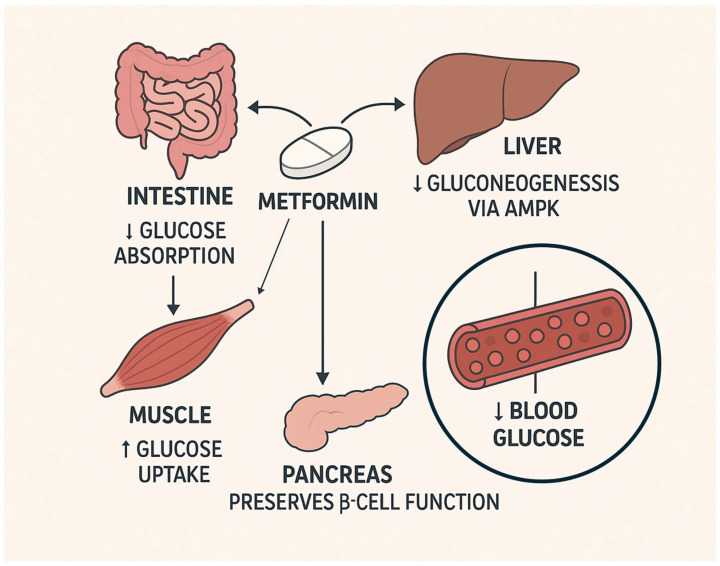
The effect of metformin. This diagram illustrates metformin’s well-established effects on several organs in T2DM. Metformin reduces glucose absorption in the intestines. In the liver, metformin activates AMPK, resulting in suppression of hepatic gluconeogenesis and reduced endogenous glucose production. In skeletal muscle, metformin enhances glucose uptake. β-Cell function in the pancreas is improved and preserved. These coordinated actions result in reduced glucose levels in the blood, reducing hyperglycemia [17].

Metformin is an oral antihyperglycemic medication that reduces hepatic glucose production and improves insulin sensitivity. Metformin, phenformin, buformin, and proguanil are biguanides [13]. Metformin is derived from the herb *Galega officinalis*, French lilac, and goat’s rue. This herb was initially used as a traditional medicine in medieval Europe. In the early 20th century, it was notably used to lower blood glucose due to its composition of guanidine [13]. Other derivatives were used to treat type 2 diabetes (T2D). Their use was discontinued due to their toxicity, high dose requirements, and modest glycemic impacts. In the 1940s, guanidine resurfaced as a treatment for influenza and malaria [13]. Proguanil was developed as an antimalarial agent. In search of more antimalarials, proguanil was further developed into metformin [13]. In 1949, Eusebio Garcia tested metformin in the Philippines. Flumamine, the name for metformin hydrochloride at the time, became an anti-influenza agent, with the side effects of lowering blood glucose levels [14]. In 1957, the French physician Jean Sterne reported the use of metformin to treat type 2 diabetes (T2D). During his research, Sterne found metformin to be the most efficacious due to its ability to lower glucose levels with minimal adverse effects and its reduced toxicity at higher dosages compared to those of other guanidine-based compounds. Sterne coined the term ‘Glucophage’ for metformin as an antihyperglycemic agent, referring to how this compound diminishes glucose levels and reduces hepatic glucose production (Figure 1) [13]. This is assisted with marketing metformin to physicians. Metformin was considered as even more desirable when it was discovered that prolonged use results in little to no physiological damage, unlike sulfonylureas. Prolonged use of sulfonylureas can result in numerous adverse effects, including insulin burnout, pancreatic fatigue, cardiovascular risk, and hypoglycemia [13].

While metformin reduces glucose production in the liver through AMPK-dependent (Figure 1) and independent pathways, it also inhibits inflammation through these same pathways [15]. AMPK is a metabolic sensor that links energy homeostasis to the regulation of inflammatory signals. An increased AMP: ATP ratio serves as an activation signal that triggers AMPK when an energy deficiency occurs [16].

### 2.1. Metformin Pharmacokinetics

Metformin is primarily administered orally. It improves glucose tolerance in T2D by lowering both basal and postprandial plasma glucose (PPG) levels. Its molecular structure is planar and hydrophilic [17]. It is monoprotonated at a neutral pH, with several tautomeric configurations [18]. The half-life of metformin is about 5 h [17]. During its time in the body, it is distributed to several tissues, including the liver, intestines, and kidneys [17].

The oral bioavailability of metformin is about 50%. This is absorbed through the upper small intestine, specifically, in the duodenum and jejunum [19,20]. The intestinal absorption of metformin is believed to be predominantly mediated by the plasma membrane monoamine transporter (PMAT) [21]. The PMAT, encoded by the SLC29A4 gene, is expressed on the enterocytes of the intestinal lumen. Metformin’s mechanism of action is still being explored and remains a debated theory [22]. Organic cation transporter 3 (OCT3), encoded by the SLC22A3 gene, is expressed on the border enterocytes. This is thought to contribute to metformin uptake [22]. OCT1, encoded by the SLC22A1 gene, is expressed in enterocytes’ basolateral membranes and cytoplasm. This is considered to facilitate the transport of metformin into the interstitial fluid. OCT1’s and OCT3’s roles in metformin’s intestinal transport remain undefined [22]. The liver is believed to be the primary site of action for metformin. This presumption is based on the higher levels presented in hepatocytes as opposed to levels in the blood [22].

Metformin is not metabolized and remains unbound and unaltered during its time in circulation [23]. Several hepatic mechanisms for metformin have been proposed. These include inhibiting glucagon-stimulated cAMP production by blocking adenylyl cyclase, activating AMPK via liver kinase B1, reducing energy charges, and increasing the AMP:ATP ratio by impairing the mitochondrial NADH–coenzyme Q oxidoreductase (complex I) of the electron transport chain (only at a 5 mM metformin concentration and higher) [20]. Another recently proposed mechanism involves reducing lactate and glycerol to glucose by inhibiting mitochondrial glycerophosphate dehydrogenase through an altered redox state, ultimately decreasing hepatic gluconeogenesis [24].

The kidneys are the primary route of elimination. The kidneys excrete 90% of the drug [23]. OCT2, which is predominantly expressed at the basolateral membrane in the renal tubules, primarily facilitates the transportation of metformin from circulation into renal epithelial cells [22]. Metformin can also be a substrate for multidrug and toxin extrusion (MATE) transporters. MATE1 and MATE2-K are expressed in the apical membrane of the renal proximal tubule cells. MATE1, encoded by SLC47A1, is highly expressed in the liver, skeletal muscle, and kidneys. It contributes to metformin excretion from the liver and kidneys. MATE1 contributes to biliary excretion, which is insignificant in humans. MATE1 and MATE2-K mediate renal metformin excretion from tubule cells to the lumen. PMAT is expressed on the apical membrane of renal epithelial cells and is believed to contribute to the renal reabsorption of metformin [22].

### 2.2. Diabetes Mellitus Pathophysiology

**Figure 2:**
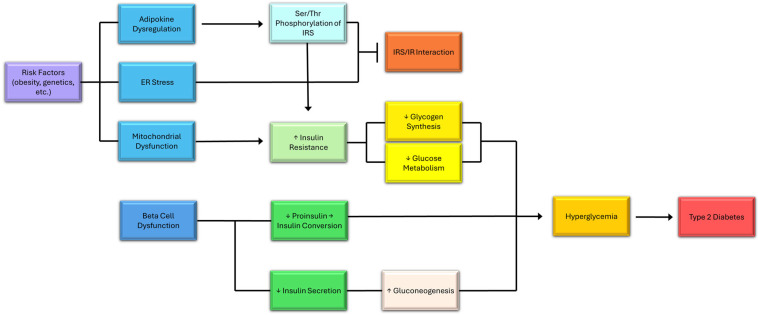
Pathophysiology of type 2 diabetes. Risk factors, like obesity and genetics, trigger a multitude of mechanisms, including adipokine dysregulation, ER stress, and mitochondrial dysfunction. Adipokines (TNF-α) can disrupt insulin signaling and trigger the Ser/Thr phosphorylation of insulin receptor substrates (IRSs). This, along with ER stress, inhibits IRS and insulin receptor (IR) interaction. These mechanisms result in increased insulin resistance, causing reduced glycogen synthesis and glucose metabolism. Dysfunctional pancreatic β-cells exhibit impaired insulin secretion and reduced proinsulin-to-insulin conversion. Reduced insulin secretion gives rise to gluconeogenesis. This deficiency in insulin production and a lack of insulin sensitivity exacerbate the imbalance between glucose and insulin, resulting in hyperglycemia, which is a major indicator of T2DM.

Type 2 diabetes mellitus (T2DM) is a condition caused by insulin resistance (Figure 2), which is defined by the failing responses of cells in the liver, adipose tissue, pancreas, hypothalamus, and skeletal muscles to insulin [25]. In this condition, the pancreas cannot produce enough insulin to control the glucose input, leading to hyperglycemia [26]. Several drugs have been developed to treat this condition, including the most notable, metformin [17]. T2DM is often implicated in neurodegeneration. It is distinguished by peripheral insulin resistance, which causes reduced hepatic glucose uptake [25].

Pancreatic β-cells are critical for maintaining the homeostasis of glucose levels. They are responsible for the synthesis, storage, and secretion of insulin. β-Cells are found in islets. Islets differ from pancreatic exocrine tissue [25]. Pancreatic exocrine tissue secretes pancreatic enzymes and fluid, which drain into the duodenum. Islets comprise various cell types, 70% of which are β-cells, localized in the islets’ cores. β-Cells are surrounded by α-cells, which secrete glucagon; δ-cells, which secrete somatostatin; and pancreatic polypeptide (PP) cells, which secrete pancreatic polypeptide [27]. Each of these cell types influences the production of the others. Glucagon production from alpha-cells is suppressed by insulin production from β-cells. Somatostatin inhibits insulin and glucagon production [28]. PP suppresses somatostatin secretion [29]. Besides insulin, β-cells also produce proinsulin, amylin, and C-peptide. In β-cells, the rough endoplasmic reticulum ribosomes synthesize preproinsulin, which is cleaved into proinsulin and transported to the Golgi apparatus. In those with T2D, these cells will become defective, resulting in heightened glucose levels. Proinsulin is packaged in secretory vesicles near the cell membrane, where it will be cleaved, creating equimolar amounts of insulin and C-peptide. β-Cell failure may become imminent in specific individuals, particularly those who do not maintain control of their condition. β-Cell destruction may be avoided through diet, regular exercise, and insulin regulation [30].

Impaired insulin secretion is considered a risk factor for T2DM [31]. β-Cell dysfunction is characterized by a dysfunction in insulin secretion (Figure 2) during glucose stimulation. This is believed to occur before the onset of glucose intolerance in type 2 diabetes mellitus (T2DM). The insulin response depends on the glucose transport and coupling to the glucose sensor. This creates a glucose/glucose sensor complex. The glucose sensor complex primarily involves glucokinase (GK) and glucokinase regulatory protein (GKRP). The complex plays crucial roles in glucose metabolism by stabilizing and activating GK in response to rising glucose levels. In both pancreatic β-cells and hepatocytes, high extracellular glucose levels promote conformational and regulatory changes that enhance GK stability and prevent its degradation. In hepatocytes, GKRP binds to and sequesters GK in the nucleus during low-glucose states, thereby inhibiting its activity. When glucose levels rise, GK is released from glucose kinase regulatory protein (GKRP), translocates to the cytoplasm, and becomes catalytically active, thereby facilitating glycolysis and glycogen synthesis. This translocation and stabilization ensure a sustained metabolic response, allowing glucose to be efficiently phosphorylated to glucose-6-phosphate. In β-cells, this mechanism contributes to ATP generation, which is essential for insulin secretion, effectively linking glucose sensing with hormonal regulation. The GK/GKRP complex increases GK by stabilizing proteins and reducing protein degradation [32]. The β-cells of T2DM patients tend to have significantly reduced glucose transport. This alters the controlled portion of insulin secretion, shifting it from glucokinase to the glucose transport system. The next phase of T2DM is known as β-cell glucotoxicity. It involves the impaired release of newly synthesized insulin [30]. This function, however, can be restored by repairing glycemic control. This desensitization is caused by glucose’s paradoxical inhibitory effect when insulin is released. This can be attributed to the amount of glycogen accumulated in the β-cell, resulting from sustained hyperglycemia [30]. β-Cell dysfunction may also result in asynchronous insulin release, reduced proinsulin-to-insulin conversion, and impaired glucose potentiation when responding to non-glucose insulin secretagogues (Figure 2) [30]. Besides the pancreas, major organs and tissues affected by insulin resistance include the liver, skeletal muscle, and adipose tissue [33].

Gluconeogenesis generates glucose from non-carbohydrate carbon sources, primarily lactate and pyruvate. This is the reverse process of glycolysis, which converts glucose to lactate or pyruvate [34,35]. Gluconeogenesis primarily occurs in the mitochondria and cytoplasm of the liver and kidneys. Insulin resistance in T2DM reduces glucose metabolism and decreases glycogen synthesis [36]. This leads to increased lipogenesis. Insulin is an inhibitor of gluconeogenesis. As a result, there will be increased gluconeogenesis in the liver [34,37].

### 2.3. Diabetes and Inflammation

Current theories explain β-cell dysfunction and insulin resistance in type 2 diabetes mellitus (T2DM) via endoplasmic reticulum (ER) stress, oxidative stress, amyloid deposition in the pancreas, ectopic deposition in the muscles, liver, and pancreas, as well as lipotoxicity and glucotoxicity [38]. These mechanisms are believed to induce, exacerbate, or contribute to inflammation [39]. Previous studies have indicated the elevated presence of cytokines and chemokines, such as interleukin (IL)-1β, IL-6, and acute-phase proteins, such as C-reactive protein (CRP) [39]. These proteins are predictive proinflammatory factors for T2DM. The serum concentration of IL-1 receptor antagonist (IL-1RA) is highly upregulated in obese and prediabetic individuals promptly before the onset of T2DM. IL-1β induces the expression of IL-1RA and counters its expression. Elevated IL-1RA levels indicate high IL-1β levels and reflect the body’s attempt to lower the cytokine level [40]. Upregulated CRP levels are a common and highly accurate indicator of cardiovascular disease in patients with T2DM, and elevated proinflammatory factors in T2DM are mostly dependent on IL-1. Therefore, several studies have concluded that IL-1 inhibition can reduce the concentrations of these factors [39].

Relying on the expression levels of IL-1β, IL-6, and CRP in T2DM is not enough to determine the measure of inflammation expressed in individual tissues. These levels may only reflect the activation of immune cells. The ratio of the pancreatic islets’ volume to the blood volume is low. An inflammatory islet measurement would not accurately depict an inflammatory response within the circulation [39]. However, measuring an inflammatory response in adipose tissue would differ significantly. Those who suffer from obesity most often have significant amounts of adipose tissue, which can account for more than half of one’s bodyweight when the condition is morbid. Additionally, the liver is a relatively large organ that produces CRP via IL-6 induction [41]. Therefore, adipose tissue and the liver largely contribute to circulating proinflammatory markers. However, IL-6 and CRP levels in circulation are insufficient to predict an anti-inflammatory response to treatment targeting insulin resistance [39]. Also, according to previous animal studies, tumor necrosis factor (TNF) was produced from the adipose tissue of obese participants [42]. This indicated early on that insulin resistance could present inflammatory consequences.

The AMP-activated protein kinase (AMPK) pathway is a target for controlling type 2 diabetes. AMPK is a nutrient-sensing serine/threonine enzyme that is a master metabolic regulator. AMPK consists of a heterotrimer, composed of catalytic α-subunits (α1 and α2), as well as regulatory β-subunits (β1 and β2) and γ-subunits (γ1, γ2, and γ3), making 12 possible isoforms [43]. It can also be triggered by the phosphorylation of Thr172 on the α-subunit of the activation loop by upstream kinases. These kinases include calcium/calmodulin-dependent protein kinase β (CaMKKβ), tumor-suppressor liver kinase B1 (LKB1), or transforming growth factor-β-activated protein kinase-1 (TAK1) [44]. AMPK activation may be inhibited at Ser485 of the α1 subunit and Ser491 of the α2 subunit [45]. The inhibitory effect of Ser485 at the α1 subunit occurs when it is autophosphorylated or phosphorylated by Akt or protein kinase A (PKA) in several cell types and tissues [46]. The inhibitory effect of Ser491 at the α2 subunit occurs when it is similarly autophosphorylated or phosphorylated by PKA or p70S6K [47]. γ-Subunits are composed of four cystathionine-beta-synthase (CBS) domains. Each pair is known as a Bateman domain. AMP and other adenine nucleotides bind to three of the four CBS domains. The first two domains can bind AMP, ADP, or ATP, depending on their relative concentrations. The third domain mainly binds to AMP and occasionally binds to ATP, depending on the situation. When the AMP:ATP ratio increases, AMP replaces the bound ATP at the Bateman domains. This enhances activation via the allosteric effect of AMP binding, assisting AMPK activation. However, AMP binding significantly reduces the rate of dephosphorylation [48]. When intracellular energy levels are low, AMPK becomes activated. When AMPK is activated, homeostatic energy levels are restored as AMPK sends signals by stimulating ATP production and inhibiting ATP usage. The observed dysregulation in AMPK signaling, contributing to insulin resistance and T2DM, has led to increased interest in this pathway [49]. The activation of AMPK has been shown to ameliorate these pathologies. Overall, AMPK activation improves glucose homeostasis and insulin sensitivity, making it an ideal target for treating T2DM [50].

## 3. The Relationship Between Neurodegeneration and Diabetes Mellitus

**Figure 3:**
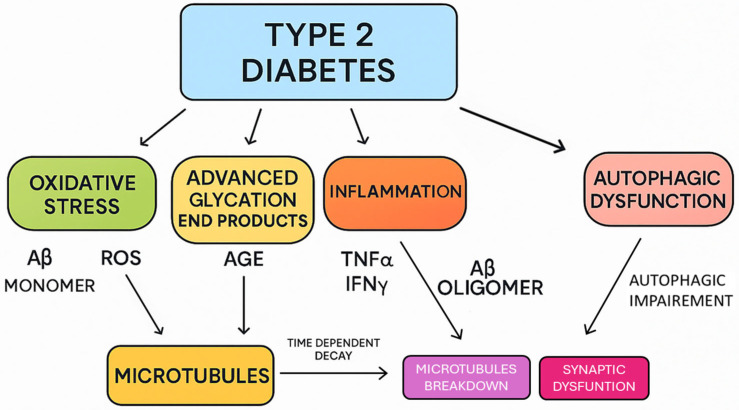
The effects of type 2 diabetes. This diagram illustrates the molecular mechanisms linking type 2 diabetes (T2DM) to neuronal microtubule damage and synaptic dysfunction. T2DM induces oxidative stress, advanced glycation end-products (AGEs), inflammation, and autophagic dysfunction. These pathways increase reactive oxygen species (ROSs), cytokines (TNFα and IFNγ), and Aβ oligomers while impairing protein clearance. The resulting stressors destabilize tau-bound microtubules, leading to the progressive breakdown of microtubules and synaptic impairment, which contribute to neurodegenerative processes.

The link between T2DM and neurodegenerative diseases underscores their overlapping pathophysiological pathways, which helps to explain why antidiabetic agents, like metformin, are being explored as potential treatments for various neurodegenerative conditions [51]. Notably, AD is often described as “type 3 diabetes” because it shares similar molecular drivers and risk factors with T2DM. As shown in Figure 3, persistent hyperglycemia in T2DM triggers neurotoxic processes, including oxidative stress, the formation of advanced glycation end-products (AGEs), and chronic inflammation. These processes mirror key mechanisms underlying neurodegeneration [52]. These metabolic imbalances damage cellular structures, promote protein misfolding and aggregation, and foster a neurochemical environment that supports neurodegenerative changes, as reflected in the significantly elevated risks of AD and PD among people with T2DM. Central insulin resistance further reinforces this connection by disrupting the brain’s insulin signaling, which is essential for synaptic plasticity, glucose metabolism, neuronal survival, and cognitive function [53]. The inflammatory interplay between T2DM and neurodegeneration is also critical, as systemic inflammation in T2DM activates microglia and sustains a neuroinflammatory state similar to that found in several neurodegenerative disorders [54,55]. Vascular complications associated with T2DM, such as microangiopathy, further impair the cerebral blood flow, compromise the BBB’s integrity, and hasten neurodegenerative progression [56,57]. Clinically, these intertwined mechanisms contribute to more rapid cognitive decline and an earlier emergence of neurodegenerative symptoms in individuals with T2DM [58,59]. This convergence of biological pathways provides a strong mechanistic basis for the hypothesis that metformin may counteract neurodegenerative processes by modulating multiple shared pathological targets, supporting efforts to repurpose this established T2DM medication for neurodegenerative disease management [60].

## 4. Neurodegeneration

Neurodegeneration is the loss of function and death of neurons in the nervous system. Common causes of neurodegeneration include chronic inflammation and oxidative stress [61]. These mechanisms may be triggered by internal or external factors, including, but not limited to, lifestyle, age, genetic, toxic, chemical, or viral factors. This results in neurodegenerative conditions and diseases, including Alzheimer’s disease (AD), Parkinson’s disease (PD), Huntington’s disease (HD), brain tumors, strokes, and several other conditions [62]. The most common risk factor for neurodegenerative disorder development, particularly AD and PD, is aging. AD and PD are, respectively, the most common neurodegenerative diseases. Also, individuals with type 2 diabetes have an increased risk of developing neurodegenerative diseases.

Neurodegenerative diseases may be classified into several categories. They may first be classified based on the anatomical location. Disorders of the central nervous system (CNS) may be localized to specific areas, including the brainstem, cerebellum, spinal cord, cerebral cortex, or basal ganglia. They may be further classified based on significant clinical features, such as dementing and non-dementing [62]. According to the Alzheimer’s Association, there are currently over 6 million people in the US who suffer from AD [63]. This number is expected to increase to 13 million by 2050. Two-thirds of those who suffer from AD in the US are women. Elderly African American and Hispanic women have the highest incidence rates of AD. However, these demographics are far less likely to receive a diagnosis for their condition when compared to Caucasian Americans, according to the Alzheimer’s Association. Alzheimer’s disease (AD) is characterized by the presence of amyloid-beta (Aβ) plaques and neurofibrillary tangles within the intracellular environment, neuronal death, and synaptic loss. These factors are major contributors to cognitive decline. The formation of abnormal neurofibrillary structures may be a result of tau phosphorylation. AD ultimately results in neuronal loss and cerebral atrophy [64].

According to the Parkinson’s Foundation, there are currently around 500,000 reported cases of PD in the US. By 2030, it is estimated that the prevalence of PD will rise to 1.2 million. Men are 1.5 times more likely to suffer from PD than women. Some studies suggest that African–American men have the highest incidence rate, while others suggest they have the lowest and Hispanic men have the highest [65]. The cause of PD is unknown, but environmental factors are suspected to be responsible [66].

**Figure 4:**
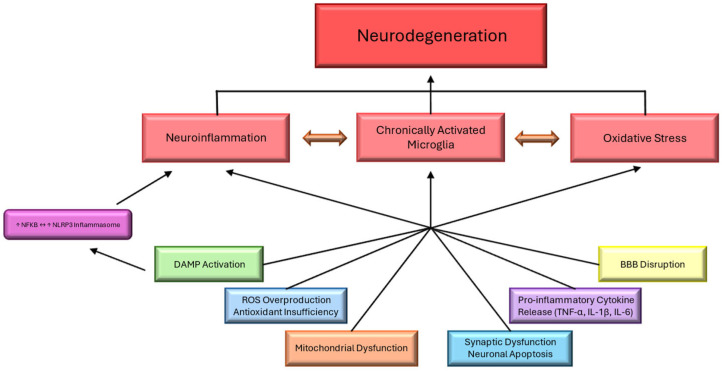
Causes of neurodegeneration. Neuroinflammation, oxidative stress, and chronically activated microglia are well-known causes of neurodegeneration.

Neurodegeneration is driven by a complex interplay of interconnected pathological processes, where diverse factors can mutually reinforce and aggravate disease progression through various feedback and feedforward cycles (Figure 1). These neuronal factors, including the blood–brain barrier’s (BBB’s) disruption, synaptic dysfunction, neuronal apoptosis, and mitochondrial dysfunction, can trigger or be triggered by others, leading to a vicious cycle that exacerbates neuronal damage and loss (Figure 4). Neuronal factors that interlink in neurodegeneration include the following:

(1) Synaptic Dysfunction: Synaptic changes, including loss and dysfunction, occur early during neurodegeneration and are intricately linked to cognitive decline, particularly in the aging population. Proinflammatory cytokines and oxidative stress can precipitate disrupted synaptic function, exacerbating neuronal vulnerability and accelerating degeneration [67];
(2) Neuronal Apoptosis: Neuronal apoptosis is a key mechanism of cell death in neurodegenerative diseases, often triggered by a convergence of factors, including proinflammatory cytokines, mitochondrial dysfunction, reactive oxygen species (ROSs), and the loss of trophic support. The progressive loss of neurons accelerates disabilities associated with conditions like Alzheimer’s and Parkinson’s diseases [68];
(3) Mitochondrial Dysfunction: Mitochondrial impairment is central to neurodegenerative pathology, driving energy failure and increased production of reactive oxygen species (ROSs) and triggering cell death pathways. Defective mitochondria enhance neuronal susceptibility to apoptosis and interact with other cellular stress sources, such as inflammation and DAMP signaling [69].

### 4.1. Blood–Brain Barrier (BBB) Disruption and Neurodegeneration

BBB disruption is both a cause and a consequence of neurodegeneration. Increased permeability allows peripheral immune cells and potentially toxic molecules to infiltrate the brain, amplifying inflammatory responses. The altered barrier environment further primes the brain for immune activation and promotes the entry of danger-associated molecular patterns (DAMPs) and cytokines, contributing to disease progression.

### 4.2. Inflammatory Signaling Pathways Involved in Neurodegeneration

The NF-κB pathway is a central regulator of inflammation in neurodegeneration. The activation of NF-κB in neurodegeneration occurs in response to oxidative stress, misfolded proteins, and infection. NF-κB induces the expressions of genes encoding proinflammatory cytokines, such as TNF-α and IL-1β. As shown in Figure 3, persistent NF-κB activation has been observed in several neurodegenerative diseases [16]. NF-κB represents a group of transcription factors and an inflammatory pathway with potential neuroprotective properties. The NF-κB pathway is composed of p50 (NF-κB1), p52 (NF-κB2), p65 (RelA), RelB, and c-Rel. These are responsible for mediating the transcription of target genes.

The canonical and noncanonical NF-κB pathways differ in signaling mechanisms but are responsible for regulating inflammatory responses [17]. When the canonical NF-κB pathway is activated, it induces the production of proinflammatory cytokines. These cytokines include TNF-α, IL-1β, and IL-6. The release of cytokines triggers an inflammatory response. The proinflammatory cytokines, in turn, further activate the canonical NF-κB pathway in a positive feedback cycle. Targeted genes are transcribed by RelA and p50 heterodimers during activation. During the steady-state activation, IκB proteins bind to and sequester RelA and p50 in the cytoplasm. IκB is an NF-κB inhibitor. It is composed of precursor proteins (p100 and p105), typical IκB proteins (IκBα, IκBβ, and IκBε), and atypical IκB proteins (IκBζ, BCL-3, and IκBNS). The IKK phosphorylation of IκB proteins is the most significant event during canonical NF-κB activation. IKKs are composed of IKKα (IKK1), IKKβ (IKK2), and IKKγ (NF-κB’s essential modulator (NEMO)) [18].

Danger-associated molecular patterns (DAMPs), such as misfolded protein aggregates, act as signals that initiate and sustain neuroimmune responses. DAMPs activate glial cells via specific receptors, amplifying innate immune responses and inflammation. At the cellular level, DAMP-dependent pathways can initiate self-perpetuating cycles that either sustain or exacerbate neurodegenerative damage. DAMPs, formed from aggregated proteins or cellular damage, can stimulate the NOD-like receptor protein 3 (NLRP3) inflammasome in microglia, resulting in robust inflammatory responses and dopaminergic neurodegeneration. The NLRP3 inflammasome is a key component of the innate immune response. Upon activation by danger-associated molecular patterns (DAMPs) and pathogen-associated molecular patterns (PAMPs), the NLRP3 inflammasome triggers caspase-1 activation, releasing cytokines IL-1β and IL-18. The dysregulation of the NLRP3 exacerbates neuroinflammation in AD and PD [70]. There exists an activation cycle: DAMPs trigger inflammasome and NF-κB activation, promoting further neuroinflammation, cell death, and the release of additional DAMPs, maintaining and amplifying neurodegeneration (Table 1).

Moreover, the JAK/STAT pathway transmits signals from extracellular cytokines, such as IL-6, to the nucleus. Abnormal activation in neurodegeneration can contribute to the production of proinflammatory cytokines. Elevated JAK/STAT signaling is reported in various neurodegenerative conditions, promoting chronic inflammation [71]. Mitogen-activated protein kinase (MAPK) pathways, including p38, c-Jun N-terminal kinase (JNK), and extracellular signal-regulated kinase (ERK), regulate inflammation, oxidative stress, and apoptosis [72]. Increased MAPK signaling has been associated with neuroinflammatory processes in AD and PD, contributing to synaptic dysfunction and neuronal loss.

Toll-like receptors (TLRs) detect endogenous danger signals. TLR activation initiates cascades that produce proinflammatory cytokines. In the central nervous system (CNS), TLR activation has been implicated in the pathogenesis of neurodegenerative diseases. TLR4 activation by amyloid-β in Alzheimer’s disease exacerbates neuroinflammation [73]. Aβ formation and abnormal tau deposition are common theories used to explain neurodegeneration. However, their roles in neuroinflammation are not fully understood. Most targeted therapies rely on the activation of caspases and transcription factors, such as NF-κB and AP-1, which, in turn, produce proinflammatory cytokines, including IL-1β, TNF-α, and IL-6 [74]. These cytokines may act on neurons to induce apoptosis. Moreover, when IL-1β and TNF-α are released from microglia, they activate astrocytes. Factors released from astrocytes may result in further microglial activation.

### 4.3. Neural Mechanisms of Alzheimer’s Disease (AD)

Alzheimer’s disease affects numerous cell types, including the hippocampus, amygdala, substantia nigra, locus coeruleus, brainstem nuclei, thalamus, hypothalamus, claustrum, and certain regions of the cerebral cortex. The types of neuronal cells affected vary according to the expression of neuromodulators, neuropeptides, and neurotransmitters. Cognitive decline in AD strongly correlates with reduced synapse density in the hippocampus and cerebral cortex [75]. Dendritic spine loss has been directly associated with AD. Past studies have demonstrated that this reduction, as a loss, is proportional to AD pathogenesis [76]. This supports a reasonable belief that an observable increase in dendritic spine loss accurately indicates disease progression [77].

### 4.4. Pathogenesis of Alzheimer’s Disease (AD)

Alzheimer’s disease (AD) is characterized by changes in neurotransmitter expression, synaptotoxicity, amyloid-beta (Aβ) protein deposit (amyloid/senile plaque) accumulation, reduced neutrophils, widespread neuronal death, and neural atrophy [78,79]. The most explored of these is the Aβ-amyloid hypothesis [80]. This theory states that the excessive production of Aβ peptide, specifically Aβ42, is responsible for AD’s neurodegenerative pathology—neurofibrillary tangles, synapse loss, amyloid/senile plaques, and neuronal death [81]. Although this theory remains popular, multiple studies have shown a weak association between Aβ deposits and AD-related neuropathology. Several Aβ conformations are formed from the proteolytic cleavage of amyloid precursor protein (APP), a type I cell-surface protein [82].

The tau hypothesis gained popularity around the same time as the Aβ-amyloid hypothesis [82]. This hypothesis describes that amino acids in tau proteins are hyperphosphorylated. This causes the proteins to dissociate from the microtubules, changing the transport structure, which results in neuronal starvation and cell death [83]. This mechanism makes hyperphosphorylated tau important for intracellular neurofibrillary changes. This supports the theory that tau phosphorylation contributes to the formation of neurofibrillary tangles in Alzheimer’s disease (AD) pathogenesis. Tau is primarily found not only in axons but also at lower concentrations in dendrites [82].

### 4.5. Neurotransmitters Affected in Alzheimer’s Disease (AD)

The primary neurotransmitters in AD pathology are acetylcholine, glutamate, GABA, serotonin, somatostatin, and dopamine. Acetylcholine (ACh) is a central and peripheral nervous system neurotransmitter [84]. The cholinergic hypothesis was one of the first proposed hypotheses to explain AD’s etiology [85,86]. The cholinergic system plays crucial roles in regulating memory, learning, stress, rapid-eye-movement (REM) sleep, attention, and other cognitive functions [84,87]. The basal forebrain is a significant source of cholinergic neurotransmitters [84]. Cholinergic neurons are key components in neuronal growth and synaptic plasticity [88]. They are primarily found in the basal forebrain, brainstem, basal ganglia, and spinal cord of the central nervous system (CNS) [89]. The use-dependent plasticity of the motor cortex in humans is obstructed by cholinergic antagonists and facilitated by acetylcholinesterase inhibitors [90]. The brain tissue of individuals with AD reveals reduced acetylcholine synthesis, decreased choline acetyltransferase (ChAT) activity, and decreased choline release and uptake, which are characteristic of cholinergic neuronal degeneration and impaired transmission [91].

ACh synthesis is catalyzed when choline acetyltransferase (ChAT) transfers an acetyl group from acetyl-CoA to choline in cholinergic presynaptic neurons [92]. ACh is stored in vesicles and broken down in the synaptic gap by acetylcholinesterase (AChE) [93]. Afterward, the vesicular acetylcholine transporter (VAChT) translocates ACh from the cytosol to the synaptic vesicles [94]. ACh release is controlled by Ca^2+^-mediated exocytosis. ACh receptors are excitatory and may be classified as nicotinic, ionotropic, muscarinic, or metabotropic. Nicotinic ACh receptors (nAChRs) are transmembrane proteins that act as cation-selective, ligand-gated ion channels. NAChRs bind ACh, leading to the opening of the channel. This diffuses Na^+^ and K^+^ ions into the cell [84]. Currently, the primary drugs used to treat AD are cholinesterase inhibitors and acetylcholinesterase inhibitors. Both drug types prevent the degradation of ACh to acetate and choline. This increases the availability of acetylcholine in the CNS [95].

On the other hand, glutamate is a major excitatory neurotransmitter in the central nervous system (CNS) and is synthesized from glucose. It is also a non-essential amino acid [96]. Previous studies have indicated a connection between altered glutamate and glucose levels with aging. Glucose use is downregulated in those with AD, which reduces their glutamate levels [97]. These alterations typically occur before the deposition of Aβ plaques. The presynaptic terminal of glutamatergic neurons contains glutamate within the synaptic vesicles [98]. Upon stimulation, glutamate is released from the vesicles and transported to the synaptic cleft. Glutamate receptors are included within the postsynaptic terminal. The receptors are transmembrane proteins that transfer the glutamate signal to the intracellular environment. Vesicular glutamate transporters transport glutamate to synaptic vesicles [98]. Astrocytes release the transporters into the synaptic cleft, leading to the presynaptic membrane reuptake of the transporters, where glutamate is converted by glutamine synthetase to glutamine [99]. There are two types of glutamate receptors: metabotropic glutamate receptors (mGluRs) and ionotropic glutamate receptors (iGluRs). MGluRs are G-protein coupled and control processes through the G-protein-signaling cascade. IGluRs are ligand-gated ion channels that produce excitatory currents and are stimulated by glutamate [97]. AMPA receptors (AMPARs) and NMDA receptors (NMDRs) are transmembrane proteins that function as ionotropic receptors. AMPARs primarily mediate CNS excitatory synaptic transmission and are the first receptors to react to glutamate. They help to depolarize postsynaptic neurons via Na^+^ influx and K^+^ efflux, which mediate quick excitatory synaptic transmission. NMDRs trigger common forms of long-term synaptic plasticity at glutamatergic synapses, which trigger influx of Ca^2+^ and Mg^2+^.

When there is an overaccumulation of glutamic acid, it becomes a potent neurotoxin. This is due to the length of time exposed, which overstimulates the postsynaptic response and increases the neuronal Ca^2+^ influx. A continuous influx is caused by the constant activation of AMPARs and NMDRs, resulting in a Ca^2+^ overload. Antidepressants and anti-anxiety medications that operate on glutamate receptors can be used to treat AD. Memantine is a voltage-dependent, non-competitive NMDA receptor antagonist. It is commonly used to treat neurotoxicity in AD. Ketamine is also a non-competitive NMDR antagonist widely used to treat depression. Previous studies have indicated its effectiveness during long-term treatments at low doses, increasing tau deposition and activity in the prefrontal cortex [84,100].

GABA is the primary inhibitory neurotransmitter in the CNS. It is synthesized by glutamic acid decarboxylase (GAD) from glutamate in the cytoplasm of presynaptic neurons [101]. GAD has two isoforms, GAD65 and GAD67, distinguished by their respective molecular weights, 65 and 67 kDa. Most of the glutamate required for GABA synthesis originates from astrocytes, which play crucial role in GABA synthesis, degradation, and enrichment. The astrocytes absorb GABA, transform it to glutamine by the enzyme glutamine synthetase, and release it extracellularly [102]. Once released, it is taken up by neurons and transformed by phosphate-activated glutaminase back to glutamate. There are two distinct types of GABA receptors: metabotropic and ionotropic. These are found on the postsynaptic membrane. GABA assists in regulating neuronal excitability and network balance. Research has revealed that when amyloid-beta plaques stimulate reactive astrocytes, they release excessive amounts of GABA into the space surrounding neurons [103]. In AD, disrupted GABAergic signaling contributes to cortical hyperexcitability, synaptic dysfunction, and cognitive decline. The loss or dysfunction of GABAergic interneurons reduces inhibitory control over excitatory circuits [104]. Emerging research suggests that restoring GABAergic tone may help to rebalance neural networks and alleviate symptoms in AD [105]. The use of GABA as a target for AD therapy is important, as it aims to correct an imbalance of inhibitory signaling inputs that impair memory [106]. In AD, reactive astrocytes release excessive GABA, creating a constant “tonic inhibition” by overactivating specific extrasynaptic receptors (α5-GABAA-Rs) in the areas that house memory [104,107]. The leading therapeutic strategy involves treatments like negative allosteric modulators (NAMs). Treatments such as these selectively reduce the sensitivity of receptors to excess GABA that has been produced. This approach is designed to stimulate neurons to restore the synaptic plasticity required for learning and memory.

## 5. Metformin and the Blood–Brain Barrier (BBB)

The blood–brain barrier (BBB) is a crucial component of the CNS. It is fundamental for maintaining the brain’s microenvironment. This complex structure regulates the movement of substances between the bloodstream and the brain, ensuring the protection of neural tissue while allowing essential nutrients to traverse the BBB. Understanding the BBB’s structure, function, and implications is crucial for advancing treatments for neurological diseases and disorders [108]. The BBB comprises endothelial cells, joined by tight junctions, lining the brain’s capillaries. The tight junctions are essential to form a selectively permeable membrane, restricting the passage of molecules and ions [108].

In addition to the CNS’s endothelial cells, the BBB comprises various cellular constituents, including astrocytes, pericytes, and neurons, that collectively form a functional neurovascular unit (NVU) [109,110]. Astrocytes are crucial for the BBB’s integrity through their end-feet, which envelop blood vessels, regulating the permeability barrier and promoting the expression of tight-junction proteins [111]. Further, pericytes are fundamental components of the NVU. They interact with endothelial cells, basal lamina, and glial cells, and have a critical role in the maintenance of the structural integrity of the BBB, and help to regulate the blood flow [112]. Our understanding of the role that neurons play in maintaining the integrity of the blood–brain barrier has evolved over the last few decades. Neurons interact with the BBB’s structure, but they are not considered as a major part of it. When inflammation occurs, neurons can release substances that cause neuroinflammation, and their activity can change the blood–brain barrier’s integrity. Moreover, glutamatergic neurons have the direct ability to modify the blood–brain barrier’s integrity via increasing glutamate levels. They may also have impacts on the expressions of the BBB’s efflux transporter gene and endothelium circadian genes [113,114,115].

The primary function of the BBB is to protect the brain from pathogens, toxins, and any other harmful substances or fluctuations in the blood. The BBB also regulates the transport of neurotransmitters, nutrients, and ions essential for brain function. The BBB is highly selective, and the mechanisms used to transport substances across it can be either active or passive. Passive diffusion facilitates the transport of small, lipid-soluble molecules. Active transporters facilitate the movement of essential nutrients. Specialized transporters and ion channels facilitate the controlled movement of such molecules between environments [116].

The major transporters within the BBB include carrier-mediated transporters, active (ATP-binding) transporters, receptor-mediated transporters, and ion channels. Carrier-mediated transporters facilitate the movement of specific molecules across the BBB by binding them and undergoing conformational changes to shuttle them across the endothelial cell membrane. These transporters can be categorized into glucose transporters (GLUTs), amino acid transporters, and nucleotide transporters. GLUT1 is responsible for transporting glucose to the brain, essential for neuronal metabolism. Amino acid transporters include L-type Amino Acid Transporter 1 (LAT1). LAT1 transports leucine, phenylalanine, and other large, neutral amino acids, which help to facilitate the production of neurotransmitters and protein synthesis. Nucleotide transporters ENT1 and ENT2 mediate the transport of nucleosides, such as adenosine and guanosine. ATP-binding cassette (ABC) transporters use energy from ATP hydrolysis for transport. P-glycoprotein (P-gp) is one of the most well-known ABC transporters. It acts as an efflux pump that ejects substances, such as drugs, toxins, and metabolic byproducts, from the endothelial cells of the BBB into the bloodstream. This helps to protect the CNS by limiting the potential accumulation of harmful compounds. P-gp’s significance comes from its role in drug resistance and its ability to impair the effectiveness of pharmacological treatments by preventing them from crossing into the CNS [117]. Other ABC transporters include the breast cancer resistance protein (BCRP) and multidrug-resistance-associated proteins (MRPs) [118]. Receptor-mediated transcytosis and the low-density lipoprotein receptor (LDLR) are receptor-mediated transporters. They selectively take up molecules through specific receptors. Through transcytosis, molecules are internalized by binding to receptors on the BBB’s endothelial cells and transported in vesicles across the cell. The LDLR mediates the transport of cholesterol and other lipids across the BBB by binding to lipoprotein particles. Ion channels facilitate the movement of ions across the BBB to maintain ionic balance. Voltage-gated ion channels enable the selective influx and efflux of ions, such as sodium, potassium, and calcium, across the BBB, thereby contributing to the maintenance of electrochemical gradients [119]. Aquaporins, another type of ion channel, facilitate water transport across the BBB, thereby assisting in maintaining the cerebrospinal fluid’s balance.

The blood–brain barrier (BBB), a selectively permeable barrier that protects the brain from potentially toxic chemicals, must be crossed for metformin to treat neurological problems effectively. The BBB presents a difficulty for drug delivery to the CNS. Many therapeutics cannot cross the BBB due to its selectivity. This stresses the importance of researching drugs and delivery systems that cross the barrier. Metformin has reportedly activated neurons and glial cells by crossing the blood–brain barrier (BBB). Recent studies have shown that metformin could transport across the BBB and activate specific neurons and neuroglia to exert neurological actions [120]. According to an in vitro co-culture BBB model study, metformin is highly permeable. In a significant portion of its brain entry, organic cationic transporters (OCTs) enable carrier-mediated active transport, while the remaining portion relies on passive diffusion [121]. Metformin can alter the integrity of the blood–brain barrier, which makes it easier for neurotherapeutic drugs to enter the brain. Other studies have also demonstrated that metformin quickly penetrates the blood–brain barrier, reaches various brain regions (like the pituitary gland and hippocampus), activates the AMPK/BDNF/PI3K pathway, and promotes neuroprotective effects [122,123]. In addition, several investigations have indicated that metformin protected the BBB’s integrity, prevented barrier disruption, and maintained tight-junction proteins [124,125]. Therefore, metformin’s potential neuroprotective benefits and therapeutic implications are highlighted by its capacity to enter the brain and interact with specific neurons and circuits. The BBB is a critical structure for maintaining homeostasis in the CNS. Any disrupted integrity of the complex is associated with the progression of various neurological conditions, including stroke, AD, and MS. AD progression is believed to be related to the accumulation of Aβ and other neurotoxins due to BBB disruption [126].

## 6. Metformin’s Activation of Nrf2 and Oxidative Stress

Reactive oxygen species (ROSs) and reactive nitrogen species (RNSs) are reactive oxidants that are regularly generated due to internal and external mechanisms. Oxidants are typically released in a controlled manner. They are important regulatory signals for cellular processes, including stress responses, inflammation, cell division, autophagy, and immune function [127,128]. The uninhibited production of oxidants can result in oxidative stress, which impairs cellular function and may contribute to toxicity, chronic inflammation, and cancer development [129]. Within cells, ROSs may be produced from various sources during their natural release or due to toxic exposure or pathological injury. These sources include the cytoplasm, cell membrane, peroxisomes, endoplasmic reticulum, and mitochondria. Mitochondria are the primary sites of ROS production, producing around 90% of the cellular ROSs [130]. The most abundant form of mitochondrial ROSs is superoxide anions. Most enzymes, like cytochrome P450 and NADPH oxidase (NOX), that use molecular oxygen (O_2_) as a substrate produce ROSs as a significant product or byproduct [131,132]. Mitochondrial ROSs are formed via oxidative phosphorylation at the electron transport chain (ETC), where O_2_ is reduced to H_2_O. During transport, electrons escape and combine with O_2_ to form superoxide (O_2_^-^) at the mitochondrial sources for superoxide and hydrogen peroxide at the flavin mononucleotide (FMN) site of Complex I and in the Q cycle of Complex III [130].

At the ETC, electrons from NADH enter the electron transport chain at Complex I, while electrons from FADH2 enter Complex II. These electrons move through ubiquinone to Complex III. Afterward, they move to Complex IV via cytochrome c, where they are ultimately transferred to molecular oxygen to form water. During this process, complexes I, III, and IV send protons into the intermembrane space, creating a gradient that fuels ATP synthesis [130]. However, electrons deviate from and react with oxygen, producing superoxide, particularly at Complexes I and III, the major ROS production sites, and Complex II. Complex III produces superoxide in the matrix and intermembrane space, while Complexes I and II only generate ROSs in the matrix [130]. To form RNSs, nitric oxide (NO) must be synthesized by NO synthase. NO reacts with superoxide, forming a stronger oxidant, the peroxynitrite anion (ONOO^−^). ONOO^−^ reacts with other molecules to form RNSs, such as nitrogen dioxide (NO_2_) and dinitrogen trioxide (N_2_O_3_) [128].

Nrf2, the transcription factor encoded by the NFE2L2 gene, can be activated by oxidative stress as the master regulator of the antioxidative response [133]. Keap1 is the Nrf2-binding protein containing two known protein domains: the BTB (bric-a-brac, tram track, and broad complex) and double glycine repeat (DGR) domains. The N-terminal region contains the BTB domain [128]. The C-terminal region contains the DGR domain, which comprises Kelch repeats. Keap1 homodimerization and binding to Cullin (Cul) 3, a scaffold protein of Nrf2 ubiquitin ligase (E3), is mediated by BTB. DGR mediates Keap1 binding with Nrf2. The intervening region (IVR), the linker region (LR), lies between these two domains and is rich in cystine residues. Keap1 resides in the cytoplasm and is broadly expressed in tissues, similar to Nrf2 [128].

Since Nrf2 mRNA is expressed without inducers, it may require a post-transcriptional mechanism for activation. Induction would require Nrf2 suppression under a basal condition and Nrf2 activation by inducers [128]. ROSs are produced naturally as byproducts of aerobic metabolism, particularly within the mitochondria during oxidative phosphorylation. While ROSs play essential roles in cell signaling and homeostasis, their overproduction can lead to oxidative stress, which is implicated in aging, cancer, cardiovascular diseases, and metabolic disorders. Under normal conditions, Complex I facilitates electron transfer from NADH to ubiquinone. When Complex I is dysregulated, electrons will leak, and ROSs will form. Metformin inhibits ROS production primarily through its action on mitochondrial Complex I of the ETC. Decreasing electron flow through the ETC reduces electron leakage and ROS generation. Other than reducing ROS production, metformin enhances antioxidative mechanisms through Nrf2 activation [134]. Metformin activates AMPK, a key regulator of cellular homeostasis. AMPK activation leads to increased expressions of antioxidant enzymes, like superoxide dismutase (SOD) and catalase. These enzymes are vital in neutralizing ROSs and mitigating oxidative damage [135].

Metformin may contribute to its observed effects on longevity and age-related conditions by modulating ROS levels and enhancing antioxidant defenses. Metformin’s ability to influence oxidative stress may be a part of the reason behind its broader therapeutic effects beyond glucose control.

## 7. Autophagic Role of Metformin

Autophagy is a cellular degradation and recycling mechanism that removes dysfunctional organelles and molecules to maintain homeostasis and proper cellular function [136]. Many studies have confirmed that the special effects of autophagy in different organs or tissues regulate severe physiological and pathological processes. The biological effects of autophagy could be beneficial or harmful in other circumstances. In most organs or tissues, autophagy has different effects, which are, as a whole, valuable for them [137]. In adipose tissue, autophagy is vital for adipogenesis (in white and brown fat tissues) [138]. Several studies have demonstrated that metformin can regulate autophagy. Previous studies have shown that metformin enhances autophagy, normalizes mitochondrial function, and reduces inflammation [139]. Herein, we will explore the autophagic role of metformin, focusing on its mechanisms, effects on aging and inflammation, experimental and clinical evidence, and broader clinical implications.

### 7.1. Mechanisms of Metformin-Induced Autophagy

Increasing evidence has indicated that numerous signaling pathways are involved in the autophagy-inducing effect of metformin. Metformin stimulates autophagy by activating several pathways, notably, the AMP-activated protein kinase (AMPK)-related signaling pathways. Once activated, AMPK inhibits the mammalian target of rapamycin (mTOR), a negative regulator of autophagy. The inhibition of mTOR signaling promotes the initiation of autophagosome formation. The activation of AMPK then contributes to cellular maintenance by promoting the degradation and recycling of damaged cellular components, such as mitochondria, which support better cellular health [137]. Metformin also enhances mitophagy by increasing the expressions of mitophagy-related genes, leading to the rapid clearance of damaged mitochondria, thereby slowing aging and improving cardiovascular health [138,140,141]. Additionally, metformin can activate chaperone-mediated autophagy, as seen in the phosphorylation of specific chaperone proteins, like Hsc70, further facilitating targeted protein degradation [142].

### 7.2. Autophagy’s Effects on Aging and Inflammation

There is a strong link between autophagy and aging. With age, autophagy naturally declines, accumulating dysfunctional mitochondria, increasing oxidative stress, and heightening inflammation, collectively called “inflammaging” [143]. Research indicates that autophagy is crucial in promoting longevity, while its inhibition accelerates aging and shortens the human lifespan [144]. Previous studies have shown a link between proinflammatory factors and autophagy. Naturally aging rats were reported to have increased expressions of proinflammatory cytokines and decreased autophagy. Metformin treatment downregulated proinflammatory cytokine expression and increased autophagy [139,145]. In this setting, metformin has been shown to enhance autophagy’s activity in immune cells (such as CD4^+^ T-cells), normalize mitochondrial function, reduce the accumulation of reactive oxygen species (ROSs), and shift inflammatory cytokine profiles, particularly Th17 pathways [146]. Additionally, improved autophagy can reduce chronic inflammation, the root cause of many age-related disorders, and slow down age-related cellular degradation. Autophagy lowers the generation of inflammatory cytokines and oxidative stress by eliminating protein aggregates and defective mitochondria, promoting healthier immune function and aging [140].

### 7.3. Experimental and Clinical Evidence of Metformin Affecting Autophagy

Numerous experimental studies have shown that metformin enhances autophagy pathways both in vitro and in vivo. For instance, in cellular models of aging and metabolic stress, metformin increased markers of autophagic flux, improved mitochondrial turnover, and reduced markers of endoplasmic reticulum stress in immune cells [147,148]. These results have been corroborated in animal studies, where metformin caused increases in autophagy in organs, like the liver and intestines, and reductions in aging-related inflammatory factors [145]. These findings suggest metformin’s benefits in age-related diseases may be mediated through its pro-autophagic effects.

### 7.4. Clinical Implications of Autophagy

Metformin’s ability to enhance autophagy has important therapeutic implications. It holds promise as a therapeutic application for conditions other than diabetes management, such as cancer, neurodegenerative diseases, and aging-related disorders [149]. Clinical trials have indicated that metformin reduces markers of inflammation and improves mitochondrial function, partly through autophagy activation [16]. Moreover, its safety profile and affordability make it an attractive candidate for repurposing as an anti-aging and anti-inflammatory agent [150]. Therefore, knowledge of its mechanisms of action can help to develop personalized therapeutic approaches and combination treatments that target autophagic pathways.

### 7.5. Metformin’s Multiple Tissue-Specific Effects Through the Modulation of Autophagy

Metformin primarily modulates autophagy to produce tissue-specific effects affecting various physiological and pathological conditions. In cancer, metformin induces autophagy-related cell death in tumor-initiated cells, supporting its potential roles in cancer prevention and therapy [151]. Regarding wound healing, metformin enhances wound healing in diabetic patients by increasing autophagy via AMPK activation and affecting hypoxia response pathways, such as by modulating HIF-1α, which promotes cellular adaptation to low-oxygen conditions [152]. In diabetic kidney disease, metformin alleviates oxidative stress and promotes autophagy through the AMPK/SIRT1-FoxO1 pathway, reducing cellular damage and potentially slowing disease progression [153]. The chronic use of metformin has been associated with improved cardiac function in diabetic models, linked to enhanced cardiac autophagy and better removal of damaged cellular components in heart tissue [154]. Metformin also helps to protect blood vessels from high-blood-sugar-induced damage by supporting the autophagy machinery in endothelial cells, showing its broader protective roles beyond glucose lowering [155]. In liver and aging tissues, metformin increases autophagy, reduces inflammation, and improves metabolic health, which is beneficial in animal models for studying diabetes and aging [145].

In summary, metformin’s autophagy regulator properties highlight its potential as a multipurpose therapeutic drug. Its ability to regulate autophagy is central to its beneficial effects in diabetes, supporting tissue repair, organ protection, and oxidative stress and inflammation reduction. By activating the AMPK pathway and inhibiting mTOR, metformin enhances autophagic flux, which reduces inflammation, oxidative stress, aging, and the progression of diseases. Future studies, such as ongoing clinical trials, will elucidate the ways in which metformin-induced autophagy modification might be incorporated into clinical practice to enhance lifespan and address many diseases.

## 8. The Neuroprotective Potential of Metformin

At the core of metformin’s mechanism of action is the critical disruption of the mitochondrial function. Specifically, metformin mildly inhibits Complex I of the mitochondrial respiratory chain, leading to a reduction in adenosine triphosphate (ATP) production [156]. This shift results in an elevated AMP:ATP ratio, which serves as a signal for energy deficiency. In response, AMPK is activated through phosphorylation by upstream kinases, most notably, liver kinase B1 (LKB1). The activation of AMPK initiates a metabolic reprogramming that shifts the cell from anabolic (energy-consuming) processes to catabolic (energy-generating) ones [157]. This metabolic shift is responsible for metformin’s well-established ability to suppress hepatic gluconeogenesis and increase glucose uptake in peripheral tissues, like skeletal muscle. However, AMPK activation has far-reaching implications, impacting key pathological processes, such as inflammatory signaling, oxidative stress regulation, and cellular maintenance pathways [158], all of which are deeply implicated in the pathogenesis of neurodegenerative disorders.

Upon activation, AMPK initiates several neuroprotective cascades. Initially, it promotes mitochondrial biogenesis by activating peroxisome proliferator-activated receptor gamma coactivator 1-alpha (PGC-1α), which enhances the expressions of genes involved in mitochondrial function and oxidative phosphorylation [159]. This is vital in neurodegenerative disorders, where mitochondrial dysfunction is a hallmark feature. Furthermore, AMPK induces autophagy by directly phosphorylating ULK1, a key regulator of autophagosome initiation, and by suppressing the mechanistic target of rapamycin Complex 1 (mTORC1), a known inhibitor of autophagy. This promotes the clearance of aggregated proteins, such as amyloid-β and hyperphosphorylated tau, which accumulate in Alzheimer’s disease.

**Figure 5:**
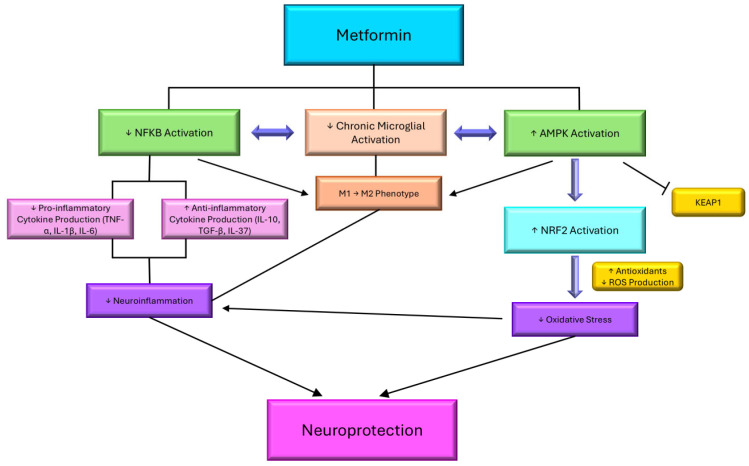
Metformin’s neuroprotective effects through microglial modulation. Metformin increases AMPK activation, lowers NF-κB activation, and reduces microglial activation. Decreasing microglial activation can reduce NF-κB activation and increase AMPK activation.

One of metformin’s most critical downstream effects is its ability to suppress chronic low-grade inflammation, a pathological feature common to both metabolic syndrome and neurodegeneration [160]. Metformin achieves this, in part, by inhibiting the NF-κB (nuclear factor-kappa B) signaling pathway, a central mediator of inflammation. Under inflammatory conditions, NF-κB drives the transcription of key proinflammatory cytokines, such as TNF-α, IL-1β, and IL-6 [161]. Through AMPK activation, metformin reduces the phosphorylation of IKKβ and stabilizes IκB, an NF-κB inhibitor, thereby preventing NF-κB translocation to the nucleus and suppressing the transcriptions of these inflammatory mediators [161]. In addition to NF-κB suppression, metformin exerts immunomodulatory effects by influencing macrophage polarization, doing so by promoting a shift from the proinflammatory M1 phenotype toward the anti-inflammatory M2 phenotype, which is associated with tissue repair and the resolution of inflammation (Figure 5) [162].

Further amplifying its anti-inflammatory action, metformin has been shown to inhibit the NLRP3 inflammasome, a cytosolic multiprotein complex that drives innate immune activation by facilitating the maturations and releases of interleukin-1β (IL-1β) and interleukin-18 (IL-18) [163]. The dysregulation of the NLRP3 inflammasome has been implicated in a range of neurodegenerative and metabolic conditions [164]. Metformin interferes with both the priming and activation steps of NLRP3 signaling. Through AMPK-dependent phosphorylation, it prevents inflammasome assembly while simultaneously reducing mitochondrial ROSs, key upstream activators of NLRP3, by stabilizing the mitochondrial function through the partial inhibition of Complex I. Moreover, by suppressing NF-κB signaling, metformin diminishes the transcriptions of inflammasome components, further limiting NLRP3 activity. Together, these mechanisms allow metformin to disrupt a central axis of inflammatory signaling. This broad capacity to dampen immune activation and promote a reparative cellular environment is particularly beneficial in neuroinflammatory contexts, where chronically activated microglia and astrocytes play key roles in driving neuronal dysfunction and degeneration.

Metformin’s role in mitigating oxidative stress, a state of imbalance between the production of reactive oxygen species (ROSs) and the capacity of the cell’s antioxidant systems to neutralize them, is equally important. Excessive ROSs, often originating from dysfunctional mitochondria, can cause lipid peroxidation, DNA damage, and protein oxidation, ultimately leading to cell death. Metformin reduces oxidative stress through multiple mechanisms. By modulating mitochondrial activity and decreasing the electron transport chain’s overactivity, it lowers the intracellular generation of ROSs at their source.

Another critical pathway influenced by metformin is the nuclear factor erythroid 2-related factor 2 (NRF2) pathway, which governs the expressions of antioxidant and cytoprotective genes. Under basal conditions, NRF2 is sequestered in the cytoplasm by KEAP1 (Kelch-like ECH-associated protein 1) and targeted for proteasomal degradation [165]. Metformin enhances NRF2 activity by inhibiting GSK-3β, which normally promotes NRF2 nuclear exclusion and degradation [166]. Additionally, mild oxidative stress induced by metformin leads to cysteine oxidation on KEAP1, allowing NRF2 to dissociate, translocate to the nucleus, and bind to antioxidant response elements (AREs) [167]. This initiates the transcription of key antioxidant enzymes, such as superoxide dismutase (SOD), catalase, glutathione peroxidase (GPx), and heme oxygenase-1 (HO-1), which, together, neutralize reactive oxygen species and restore the redox balance [168]. By promoting these detoxifying responses, metformin protects neurons against oxidative-stress-induced apoptosis and DNA damage. Moreover, NRF2 activation contributes to the phenotypic shift of microglia from a proinflammatory M1 state to an anti-inflammatory M2 state, thereby reducing the release of neurotoxic cytokines and promoting tissue repair (Figure 5).

The neuroprotective potential of metformin arises from the integration of these anti-inflammatory and antioxidant mechanisms. In models of Alzheimer’s and Parkinson’s diseases, metformin has been shown to reduce neuroinflammation by lowering the central nervous system’s cytokine levels, enhancing BDNF levels, and inhibiting glial activation [60]. Simultaneously, its enhancement of NRF2-mediated antioxidant defenses confers resistance to oxidative-stress-induced neuronal apoptosis, which is a major contributor to cognitive decline. Furthermore, AMPK activation by metformin stimulates autophagy, a critical cellular process that degrades and recycles damaged proteins and organelles. This is particularly relevant in neurodegenerative diseases characterized by the accumulation of toxic aggregates, such as amyloid-beta and alpha-synuclein [169]. By restoring the autophagic flux, metformin aids in the clearance of these neurotoxic species, thereby alleviating cellular stress and preserving neuronal function.

Beyond preservation, metformin may also contribute to the regeneration of neural tissue. Some preclinical studies suggest that metformin promotes neurogenesis, particularly in the hippocampus, by activating AMPK-dependent pathways involved in neural stem cell proliferation and differentiation [170]. If confirmed in clinical studies, this property could add a restorative dimension to metformin’s neurotherapeutic profile, particularly in diseases marked by neuronal loss and cognitive impairment.

In parallel, metformin inhibits the mTOR pathway, a nutrient-sensing and growth-regulating pathway that is often dysregulated in neurodegenerative conditions. MTORC1 promotes protein synthesis and inhibits autophagy, contributing to the accumulation of neurotoxic aggregates. Metformin suppresses mTORC1 activity through both AMPK-dependent and AMPK-independent mechanisms. AMPK phosphorylates TSC2 and raptor, key inhibitors of mTORC1, while AMPK-independent effects involve the upregulation of REDD1, a known negative regulator of mTORC1 [171]. The inhibition of mTORC1 restores the autophagic flux and reduces the buildup of dysfunctional organelles and aggregated proteins. Moreover, the hyperactivation of mTOR is associated with increased mitochondrial ROS production due to elevated metabolic activity. By attenuating mTOR signaling, metformin indirectly reduces oxidative stress and limits ROS-mediated cellular damage. Additionally, mTOR suppression has been shown to mitigate glial senescence and the senescence-associated secretory phenotype (SASP), thereby limiting chronic neuroinflammation.

Preclinical studies have demonstrated metformin’s effectiveness across various models of neurodegeneration. In Alzheimer’s models, metformin has been shown to reduce amyloid-β deposition, inhibit tau hyperphosphorylation, and improve synaptic integrity [172]. In Parkinson’s disease models, it protects dopaminergic neurons in the substantia nigra and mitigates mitochondrial dysfunction [173]. In models of vascular cognitive impairment, metformin improves cerebral blood flow, stabilizes the blood–brain barrier (BBB), and reduces the extent of ischemic injury [174]. These findings underscore the drug’s versatility and highlight its potential to slow or reverse the progression of multiple neurological disorders.

## 9. The Potential Risks of Metformin Use as a Neuroprotective Agent

Regarding cognitive function, several studies have suggested beneficial properties of metformin. Metformin may reduce the risk of cognitive decline in diabetic patients, improve cognitive function and memory, and produce antidepressant effects [60]. It has been associated with improved cognitive performance and a reduced risk of dementia. Additionally, long-term use has been linked to increased white matter integrity and reduced diabetes-related brainvolume loss, particularly in regions responsible for memory processing and spatial orientation [175].

Contrary to research studies demonstrating the beneficial effects of metformin, some research studies report the insignificant impact of metformin on cognitive function, or even a negative effect [176]. Chronic metformin treatment in normoglycemic mice impaired cognitive function, as evidenced by deficits in delayed spatial memory and discriminated avoidance learning [175]. Some research indicates that the cognitive benefits of metformin may be time-limited, diminishing, or possibly reversing with long-term use. Additionally, some studies found no significant cognitive benefits from metformin treatment in dementia prevention. A dose-related correlation was observed, where lower doses were linked to a reduced risk of dementia, but higher doses showed no benefit [176].

Metformin significantly affects brain bioenergetics [175]. In normoglycemic mice, it decreased ATP levels and activated the AMPK pathway in the hippocampus. It inhibited oxidative phosphorylation and elevated glycolysis by inhibiting mitochondrial glycerol-3-phosphate dehydrogenase (mGPDH) in vitro at therapeutic doses. This metabolic reprogramming shifts brain bioenergetics from efficient mitochondrial oxidative phosphorylation to less efficient glycolysis, potentially reducing the overall ATP production rate in vivo. At high doses (2 mM and 20 mM), metformin inhibited Complex I’s activity of isolated brain mitochondria and reduced the oxygen consumption rate (OCR) in primary astrocytes, leading to significant cell death and reduced ATP production [175]. However, at clinically relevant doses (20 µM and 200 µM), after prolonged treatment (24 h), it also reduced the OCR but surprisingly led to a significant increase in ATP production, which was attributed to enhanced glycolysis. While metformin’s general mechanism involves AMPK activation and improved energy metabolism in peripheral tissues, its impact on the brain’s metabolism is complex and potentially paradoxical [60]. Under normoglycemic conditions, it can decrease the overall ATP levels and shift the neuronal metabolism toward less efficient glycolysis. This indicates that the brain, with its high and specific energy demands, might respond differently to metformin’s metabolic reprogramming compared to other tissues, potentially leading to energy deficits or altered neuronal function under certain conditions, particularly in non-diabetic individuals or those with already compromised neuronal bioenergetics. This “metabolic paradox” implies that metformin’s neuroprotective effects are not universally applicable and are highly context-dependent [177]. It raises concerns that in certain patient populations, like non-diabetics or those with pre-existing mitochondrial dysfunction, metformin could inadvertently worsen the neuronal energy status, making its application for neuroprotection a delicate balance that requires a precise understanding of individuals’ brain bioenergetics.

A significant concern arises regarding metformin’s potential for increased amyloid-beta aggregation in specific models. Despite its proposed role in inhibiting protein aggregation, including amyloid-beta and tau, metformin has been shown to upregulate BACE1 (β-site amyloid precursor protein-cleaving enzyme 1), the key enzyme that produces Aβ from its precursor protein [175]. In one study, researchers demonstrated that therapeutic doses of metformin in mice led to increased expression of BACE1 and a corresponding rise in Aβ levels in the brain [175,178]. These rising levels have been indicated as neurotoxic. The same study observed that metformin-treated animals had greater accumulation of Alzheimer-type amyloid peptides [175,179,180]. More recent research has reinforced these findings. For instance, a 2024 translational study found that long-term metformin administration in a transgenic AD mouse model elevated soluble Aβ oligomers and the plaque burden, in conjunction with higher BACE1 expression levels [181]. This suggests that rather than protecting against amyloid pathology, metformin might *accelerate* it under certain conditions. Consistently, metformin also activated glycogen synthase kinase-3β (GSK3β) in these AD mice [181]. GSK3β is a major kinase responsible for tau protein hyperphosphorylation. Furthermore, one study found that metformin impaired learning and memory in older Alzheimer’s disease (AD)-model mice and increased amyloid pathology and tau protein phosphorylation, directly contradicting its purported benefits in Alzheimer’s disease models [176]. Chronically metformin-treated AD mice showed increased levels of phosphorylated tau and even worsened memory performance relative to those of untreated controls [181]. Such results imply that metformin could aggravate tau pathology, potentially via the overactivation of AMPK and downstream GSK3β, since excessive AMPK activity has been linked to tau aggregation in other models [181].

Beyond amyloid and tau, metformin might affect synaptic health. Research indicates that metformin can cause synaptotoxic effects in vitro. Primary neurons exposed to metformin exhibited reduced dendritic spine density, similar to the effects of Aβ toxicity. This was mediated by CaMKK2-dependent AMPK overactivation [181,182]. The loss of dendritic spines and synapses is a hallmark of cognitive decline, suggesting another mechanism by which metformin could impair neural circuits if misused over long durations. There is also evidence that metformin-induced mitochondrial dysfunction can promote protein aggregation. However, these harmful effects often depend on certain contexts. Some studies find that metformin worsens pathology in male AD-model mice but not females or only in older subjects but not younger ones [175,176,181]. These varying circumstances suggest that metformin’s interaction with neurodegenerative pathways may vary with sex hormones, age, or disease state. Nonetheless, the possibility of accelerating amyloid/tau pathology and synaptic loss raises hesitation toward the chronic use of metformin as a preventive therapy for neurodegeneration. It also urges the need for further research. It can be inferred that in certain patient populations or at specific disease stages, metformin could potentially accelerate the progression of neurodegenerative diseases by worsening core pathological hallmarks. This underscores the urgent need for highly precise patient stratification and the identification of specific biomarkers to predict who might benefit versus who might be harmed, making broad application for neuroprotection highly risky.

A study reported that diabetic patients on metformin had a higher prevalence of cognitive impairment than those not on metformin [183]. While other factors might influence this finding, it aligns with the notion that metformin’s positive effects on cognition are not universal. Another analysis noted contradictory results across studies, with specific trials showing improved cognitive test scores in metformin-treated patients, while others showed no effect or even a decline [176,184]. Overall, these mixed outcomes underscore that the long-term use of metformin for neuroprotection remains a matter of controversy. Factors such as the patient’s metabolic status, age, sex, and supplemental therapies likely modulate the cognitive and neurological effects of metformin.

Long-term metformin use can cause folate deficiency and vitamin B12 deficiency, leading to neurological damage [185,186]. Via AMPK activation and mitochondrial inhibition, metformin can induce metabolic and neurological molecular changes linked to cognitive impairment and neurodegenerative pathology, specifically, PD [175,181]. These risks do not imply that metformin is broadly neurotoxic in all cases. Instead, they highlight the need for a nuanced and monitored approach if metformin is to be used chronically for brain health.

## 10. Metformin’s Effects on Lipids and Cholesterol Metabolism

Metformin significantly affects lipid metabolism by activating AMP-activated protein kinase (AMPK), a central regulator of cellular energy homeostasis [187]. AMPK activation inhibits acetyl-CoA carboxylase (ACC) and HMG-CoA reductase, key enzymes in fatty acid and cholesterol synthesis. Through these actions, metformin has been shown to exhibit several mechanisms.

Metformin reduces hepatic lipid accumulation by promoting fatty acid oxidation and inhibiting de novo lipogenesis [188]. Also, metformin activates AMPK, which phosphorylates and inhibits acetyl-CoA carboxylase (ACC), reducing malonyl-CoA levels. This reduction alleviates the inhibition of carnitine palmitoyltransferase 1 (CPT1), a crucial enzyme that facilitates the transport of fatty acids to mitochondria for β-oxidation [189]. Concurrently, metformin suppresses the sterol regulatory-element-binding protein-1c (SREBP-1c), a transcription factor that upregulates lipogenic genes, thereby decreasing fatty acid synthesis [190]. Additionally, metformin has been shown to downregulate fatty acid synthase (FAS), further inhibiting lipid accumulation [191]. This dual action not only reduces hepatic lipid accumulation but also prevents lipotoxicity, which can contribute to neuroinflammation and neurodegenerative diseases, such as Alzheimer’s disease [192].

Metformin decreases circulating triglycerides and low-density lipoprotein (LDL) cholesterol levels, thus improving lipid profiles [189]. Metformin achieves this by activating AMPK, which inhibits the expressions of SREBP-1c and SREBP-2, key transcription factors involved in lipid biosynthesis and cholesterol homeostasis [190]. By downregulating these factors, metformin reduces hepatic triglyceride accumulation and cholesterol synthesis while simultaneously promoting LDL receptor (LDLR) expression, thereby enhancing the clearance of circulating LDL cholesterol [188,193]. Additionally, metformin suppresses diacylglycerol acyltransferase (DGAT), an enzyme responsible for triglyceride synthesis, further contributing to its lipid-lowering effects [191,194]. These mechanisms collectively improve lipid profiles, reduce atherosclerosis risk, and potentially mitigate neuroinflammatory responses in the brain that contribute to AD pathology [195,196].

Metformin enhances mitochondrial respiration and ATP production and helps to maintain neuronal energy levels, which are essential for proper neuronal function and survival [189]. Lipid peroxidation, a process where free radicals attack lipids in cell membranes, leads to cellular damage and contributes to neurodegeneration. Metformin exhibits antioxidant properties by reducing the production of reactive oxygen species (ROSs) and enhancing the activities of antioxidant enzymes [197]. By mitigating oxidative stress, metformin decreases lipid peroxidation, thereby preserving neuronal cell membranes’ integrity and preventing cell death [198].

Neural cholesterol metabolism is intricately linked to neuronal function and AD pathology. Cholesterol is crucial in synaptic plasticity, membrane integrity, and myelin formation [199]. However, excessive cholesterol accumulation in neurons can promote Aβ aggregation and plaque formation. Metformin may modulate cholesterol metabolism through several mechanisms.

Metformin downregulates sterol regulatory-element-binding proteins (SREBPs), transcription factors that regulate gene expression in cholesterol and fatty acid synthesis [190]. SREBPs are activated in response to low intracellular cholesterol levels and promote the transcriptions of HMG-CoA reductase and fatty acid synthase, critical for lipid biosynthesis. Through AMPK activation, metformin inhibits SREBP maturation and nuclear translocation, thereby reducing lipogenesis and cholesterol accumulation in hepatocytes and neurons. This regulation helps to maintain lipid homeostasis, potentially reducing amyloid-beta aggregation and neuronal lipid dyshomeostasis associated with Alzheimer’s disease [193,194].

Through AMPK activation, metformin increases the expression of ATP-binding cassette transporter A1 (ABCA1), a key transporter involved in cholesterol efflux and apolipoprotein E (ApoE) function, which is critically implicated in AD progression [195]. ApoE, particularly its ε4 allele, is a significant genetic risk factor for AD, as it is associated with impaired cholesterol transport and increased amyloid-beta (Aβ) aggregation in the brain. Reduced ApoE-mediated lipid transport can lead to synaptic dysfunction, neuroinflammation, and impaired clearance of toxic protein aggregates, all contributing to AD pathology. By promoting ABCA1-mediated cholesterol efflux, metformin may enhance ApoE lipidation, improving its roles in neuronal repair, synaptic maintenance, and Aβ clearance, thus potentially mitigating AD progression [200].

Oxidized cholesterol derivatives, such as 27-hydroxycholesterol, contribute to neuroinflammation by activating microglia and astrocytes, releasing proinflammatory cytokines, such as TNF-α, IL-1β, and IL-6. These inflammatory processes can exacerbate synaptic dysfunction and neuronal loss, which are hallmarks of AD pathology. Additionally, these oxidized cholesterol metabolites can impair the blood–brain barrier’s integrity, facilitating the entry of peripheral immune cells that further amplify neuroinflammation. Metformin’s anti-inflammatory effects, mediated through AMPK activation, help to suppress NF-κB signaling and reduce the production of inflammatory mediators, thereby potentially mitigating these harmful lipid byproducts and their neurotoxic consequences [201,202].

Several epidemiological and experimental studies suggest that metformin may exert neuroprotective effects that could be relevant for AD prevention and treatment. Insulin resistance in the brain has been implicated in AD pathology, often referred to as “type 3 diabetes” [203]. Metformin improves insulin signaling, which may help to reduce tau hyperphosphorylation and Aβ deposition [204].

Metformin activates AMPK and inhibits mammalian rapamycin (mTOR) targets, leading to enhanced autophagy. This process facilitates the clearance of misfolded proteins and toxic aggregates, including Aβ and hyperphosphorylated tau [205]. Chronic inflammation and oxidative stress are major contributors to AD pathogenesis. Metformin suppresses proinflammatory cytokines and enhances mitochondrial efficiency, reducing neuronal oxidative damage. Metformin has been shown to improve endothelial function and reduce the blood–brain barrier’s (BBB’s) disruption, which may protect against vascular contributions to cognitive impairment [118].

## 11. Metformin and Cognitive Function

Metformin has gained increasing interest for its potential benefits beyond glycemic control, particularly in cognition, Alzheimer’s disease, aging, and stroke management. As explained below, evidence from both clinical and preclinical studies indicates a complex and multifaceted impact, with benefits varying among populations and depending on underlying disease conditions.

Cognitive decline is a profound concern associated with neurodegenerative diseases. Conditions such as Alzheimer’s disease and Parkinson’s disease lead to progressive neuronal degeneration, resulting in significant impairments in cognitive function. The mechanisms of cognitive decline in these disorders are crucial for developing effective management strategies and improving patient outcomes.

The mechanisms underlying cognitive decline in neurodegeneration are multifaceted. One of the primary factors is neuronal loss, specified according to the disorder. In AD, cholinergic neurons in the basal forebrain, which play critical roles in memory and learning, are primarily affected. The loss of these neurons leads to significant deficits in cognitive abilities [206].

Abnormal protein accumulation is indicative of many neurodegenerative conditions. In AD, it is a common belief that the accumulation of amyloid-beta plaques is responsible for disrupting normal neuronal function and communication [207]. Similarly, alpha-synuclein aggregation, linked to PD, harms neuronal health and contributes to cognitive impairment [208]. This protein misfolding and aggregation hinders synaptic transmission, exacerbating cognitive decline.

Synaptic dysfunction also plays a crucial role in cognitive impairment. The loss of synapses, essential for communication between neurons, is a common feature of neurodegenerative diseases. Studies have shown a direct correlation between synaptic loss and cognitive decline, indicating the importance of synaptic integrity in maintaining cognitive function [209].

Cognitive decline manifests in various forms, affecting multiple cognitive domains, such as memory, language, and motor control. Memory loss is often one of the earliest signs of cognitive impairment, particularly in AD, where individuals struggle with both short-term and long-term memories [210]. Executive dysfunction is another common issue, particularly in PD, where individuals may find it challenging to plan, organize, and execute tasks.

While there is currently no cure for most neurodegenerative diseases, several strategies can help to manage cognitive decline. Cognitive rehabilitation, through targeted cognitive exercises, can enhance specific cognitive functions. Medications, such as cholinesterase inhibitors and memantine, are often used to manage symptoms in AD, temporarily stabilizing cognitive function. Lifestyle modifications also play a significant role in managing cognitive decline. Engaging in regular physical activity, maintaining a healthy diet, and participating in cognitive exercises can contribute to overall brain health and may slow the progression of cognitive impairment [78].

Cognitive decline in neurodegenerative diseases presents significant challenges for affected individuals and their families. Understanding the underlying mechanisms and manifestations of cognitive impairment is essential for developing effective management strategies and improving quality of life. Continued research into the processes of neurodegeneration will hopefully lead to new therapeutic approaches aimed at managing cognitive decline, offering hope for individuals facing these devastating conditions.

## 12. Emerging Potential of Metformin in Stroke Therapy

Metformin has recently garnered attention for its potential neuroprotective effects in stroke therapy. Stroke remains one of the leading causes of morbidity and mortality worldwide, necessitating the exploration of novel therapeutic avenues. Ischemic stroke, which constitutes approximately 87% of all stroke cases, results from an obstruction in a blood vessel supplying the brain, leading to neuronal injury and cell death [211]. Metformin’s potential in mitigating this damage offers a promising adjunct to existing stroke therapies.

Stroke is a leading cause of disability and mortality worldwide, primarily resulting from ischemic or hemorrhagic vascular events that lead to brain tissue damage. Recent research has identified metformin, a widely used antidiabetic drug, as a promising therapeutic agent in stroke treatment due to its neuroprotective, anti-inflammatory, and metabolic regulatory effects [212]. This article explores metformin’s emerging role in stroke therapy, emphasizing its molecular mechanisms and potential clinical applications.

Metformin is a potent AMPK activator. AMPK activation reduces neuronal apoptosis, enhances mitochondrial function, and promotes neuronal recovery following ischemic stroke [213]. Metformin mitigates ischemia-induced damage by maintaining the cellular energy balance and supporting brain repair mechanisms.

Reducing oxidative stress is an essential mechanism for neuroprotection. Oxidative stress plays a crucial role in the pathophysiology of ischemic stroke. Metformin enhances the activities of antioxidant enzymes, such as superoxide dismutase (SOD) and catalase, which neutralize ROSs. Additionally, metformin reduces the mitochondrial production of ROSs, thereby decreasing cellular damage and apoptosis [214].

Oxidative stress is a major contributor to neuronal injury in stroke. Metformin enhances the activities of SIRT1 and SIRT3, leading to improved mitochondrial function and reduced ROS production [215]. This antioxidant effect helps to protect neurons from ischemic damage and supports post-stroke recovery.

Another mechanism involves reducing inflammation. Inflammation is a key contributor to neuronal damage following a stroke. Metformin inhibits the activation of NF-κB, a transcription factor that regulates the expressions of proinflammatory cytokines, such as tumor necrosis factor-alpha (TNF-α) and interleukin-6 (IL-6). By reducing these cytokines, metformin helps to attenuate the inflammatory response and protects neuronal cells from further damage [216].

Neuroinflammation plays a significant role in stroke pathology, contributing to secondary brain injury. Metformin exerts anti-inflammatory effects by inhibiting the NF-κB signaling pathway, reducing the release of proinflammatory cytokines, such as TNF-α and IL-6 [213]. Furthermore, it suppresses the NLRP3 inflammasome, a key driver of neuroinflammation, thereby reducing stroke-induced neurotoxicity [217].

Promoting neurogenesis is an additional mechanism. Neurogenesis, the process of generating new neurons, is impaired after a stroke. Metformin activates AMPK, which upregulates the brain-derived neurotrophic factor (BDNF). The BDNF is critical in promoting new neurons’ survival, growth, and differentiation. Furthermore, metformin enhances the proliferation of neural stem cells in the hippocampus, contributing to improved cognitive function and recovery [174].

Metformin has been shown to induce autophagy, a crucial process for cellular repair and survival under stress conditions. Metformin promotes autophagy and facilitates the clearance of damaged proteins and organelles, enhancing neuronal survival [218]. Additionally, metformin stimulates neurogenesis by increasing the expression of the brain-derived neurotrophic factor (BDNF), which is essential for post-stroke neural plasticity and functional recovery [123].

Improving the blood–brain barrier’s integrity is essential. The integrity of the blood–brain barrier (BBB) is often compromised following a stroke, leading to further neuronal injury. Metformin helps to maintain the BBB’s integrity by reducing the apoptosis of endothelial cells, which form the barrier, and by enhancing the expression of tight-junction proteins, like occludins and claudin-5. This preservation of the BBB’s integrity prevents the infiltration of harmful substances and cells into the brain, reducing secondary damage [219].

## 13. Emerging Potentials of Metformin in Alzheimer’s Therapy

Alzheimer’s disease (AD) is a progressive neurodegenerative disorder characterized by cognitive decline, memory loss, and neuronal dysfunction [220]. Currently, no cure exists, and treatment options primarily focus on symptom management rather than disease modification. However, recent research has highlighted the potential to repurpose existing drugs to target AD pathology. Metformin has garnered interest due to its neuroprotective effects [221].

While metformin primarily treats type 2 diabetes mellitus (T2DM), its mechanisms extend beyond glycemic control. Several mechanisms have been proposed for AD’s therapeutic treatment, including regulating insulin signaling. Insulin resistance in the brain is increasingly recognized as a contributing factor to AD pathology. Metformin enhances insulin sensitivity and activates AMPK, which is crucial in the cellular energy balance and neuroprotection [204]. Chronic neuroinflammation, mediated by activated microglia and astrocytes, exacerbates AD pathology. Metformin has been shown to inhibit proinflammatory cytokines, thereby reducing neuroinflammatory responses [222]. Impaired autophagy is a hallmark of AD, leading to the accumulation of amyloid-beta (Aβ) plaques. Metformin enhances autophagic pathways, promoting the clearance of toxic protein aggregates [142]. Mitochondrial dysfunction contributes to neuronal damage in Alzheimer’s disease (AD). Metformin improves mitochondrial function, reduces oxidative stress, and enhances cellular energy metabolism, possibly protecting neurons from degeneration [223].

## 14. Emerging Potentials of Metformin in Aging

Aging is an inevitable biological process characterized by gradual declines in the function and integrity of an organism’s cells, tissues, and organs. It is a multifaceted phenomenon influenced by genetic, environmental, and lifestyle factors. It is often viewed because of the cumulative damage to cellular structures over time. The study of aging, or gerontology, seeks to understand the mechanisms underlying this process and to explore potential interventions that may slow down or reverse age-related decline.

Oxidative stress, resulting from an imbalance between the productions of reactive oxygen species (ROSs) and antioxidants, has long been associated with aging. ROSs are byproducts of cellular metabolism and can damage cellular components, including proteins, lipids, and DNA. Over time, this oxidative damage accumulates, leading to a gradual decline in cellular function [224]. At the cellular level, aging is associated with the accumulation of molecular damage. One of the primary theories of aging is the free-radical theory. This theory states that the accumulation of damage caused by ROSs, highly reactive molecules generated during cellular metabolism, leads to oxidative damage in DNA, proteins, and lipids. Over time, this damage impairs cellular function and accelerates aging [225].

Despite the inherent mechanisms to neutralize ROSs, including antioxidant enzymes, like superoxide dismutase and catalase, the capacity to counteract oxidative damage diminishes with age. This contributes to the progressive decline in tissue function and the onset of age-related diseases, such as neurodegenerative and cardiovascular diseases [226]. Recent studies have shown that manipulating antioxidant pathways can extend the lifespan in model organisms, providing further support for the role of oxidative stress in aging [227].

The role of genetics in aging has been central to several theories, most notably, the Programmed Aging Theory. According to this theory, aging is controlled by the genes that regulate growth and development, leading to the eventual deterioration of cellular function as a part of an evolutionary program [228,229]. Some genes are thought to govern aging-related processes, with specific mutations leading to shorter or longer lifespans in model organisms. One of the most studied examples is the roles of the telomeres, the protective caps at the ends of chromosomes that play crucial roles in maintaining genomic stability and regulating cellular lifespan. Telomere shortening is a significant contributor to aging. They comprise repetitive DNA sequences and associated proteins that protect chromosomes from degradation and fusion during cell division [230]. Telomere shortening is a hallmark of cellular aging. As somatic cells divide, telomeres progressively shorten due to the inability of DNA polymerase to replicate the ends of chromosomes completely, a phenomenon known as the end replication problem [231]. When the telomeres reach a critical length, cells could undergo apoptosis or enter senescence, a deterioration process with age. This process is crucial in the contexts of cancer, cardiovascular disease, and age-related neurodegeneration, where telomere shortening has been implicated in the pathophysiology of conditions such as Alzheimer’s disease (AD), Parkinson’s disease (PD), and Huntington’s disease (HD). Telomerase is an enzyme that extends telomeres. It has been studied for its potential to delay the onset of aging and age-associated diseases, though its application in humans is still under investigation.

Cellular senescence is when cells cease to divide but do not undergo programmed cell death. Senescent cells accumulate with age and contribute to age-related dysfunction by secreting inflammatory factors, known as the senescence-associated secretory phenotype (SASP). This accumulation of senescent cells has been linked to several age-related diseases, including cardiovascular disease, osteoarthritis, and neurodegeneration [232]. Senescent cells fail to divide, contributing to the decline in tissue regeneration and repair and leading to functional deterioration.

One of the key drivers of cellular senescence is DNA damage. Over time, cells accumulate genetic mutations due to various stressors, including radiation, environmental toxins, and metabolic byproducts. These DNA mutations impair the ability of cells to divide properly and repair damaged tissues. The p53 tumor suppressor pathway assists in inducing senescence in response to DNA damage, but prolonged or excessive activation of this pathway can accelerate aging by increasing the number of senescent cells in tissues [227].

In the brain, where many neurons do not undergo regular cell division, telomere attrition is less pronounced in individual neurons. However, neural stem cells (NSCs) in regions such as the hippocampus and subventricular zone undergo cellular division [233]. Their telomere shortening over time can limit neurogenesis, the process by which new neurons are generated. This reduction in neurogenesis has been linked to cognitive decline in aging and neurodegenerative diseases [234].

In addition to molecular damage, aging involves systemic changes, including decreased regenerative capacity and a weakened immune response. Stem cells, crucial for tissue repair and regeneration, become less effective with age. The immune system also becomes less efficient, increasing susceptibility to infections, cancer, and autoimmune diseases.

Environmental factors, such as diet, exercise, and exposure to toxins, also play critical roles in aging. For instance, caloric restriction has been shown to extend lifespan in various organisms, potentially by reducing oxidative stress and promoting cellular repair mechanisms. Regular physical activity and a balanced diet rich in antioxidants and essential nutrients have been linked to improved health outcomes in older adults, supporting the idea that lifestyle choices can influence aging [235].

Metformin influences aging through several interconnected pathways, primarily activating AMPK, modulating mitochondrial function, and reducing inflammation.

One of metformin’s primary mechanisms is the activation of AMPK, a cellular energy sensor that regulates metabolism and promotes longevity [187]. AMPK activation enhances insulin sensitivity, reduces glucose production in the liver, and promotes autophagy—a process that removes damaged cellular components, thereby improving cellular function and reducing age-related decline [236]. Mitochondrial dysfunction is a hallmark of aging, contributing to increased oxidative stress and cellular damage [237]. Metformin mildly inhibits Complex I of the mitochondrial electron transport chain, reducing ROS production [238]. Lower ROS levels decrease oxidative damage to DNA, proteins, and lipids, potentially slowing aging (Table 1).

Chronic inflammation accelerates aging and contributes to various age-related diseases, such as cardiovascular disease and neurodegeneration [239]. Metformin has been shown to lower systemic inflammation by inhibiting the NF-κB pathway and reducing levels of proinflammatory cytokines [240]. This anti-inflammatory effect may contribute to improved longevity and reduced incidence of age-related diseases.

Cellular senescence, the state in which cells stop dividing and secrete inflammatory factors, is a key driver of aging [241]. Metformin has been found to reduce senescent cell accumulation by modulating the mTOR pathway and enhancing autophagy [242]. Additionally, metformin influences sirtuins, a family of NAD^+^-dependent proteins involved in cellular stress responses, metabolism, and longevity. Specifically, metformin enhances the activities of SIRT1 and SIRT3, primarily by increasing NAD^+^ levels through the activation of AMPK. SIRT1 plays key roles in improving insulin sensitivity, reducing inflammation, and promoting mitochondrial function, while SIRT3 enhances mitochondrial resilience and resistance to oxidative stress. Metformin improves metabolic health, reduces cellular senescence, and potentially extends the human lifespan by modulating these pathways. These effects position metformin as a promising candidate for targeting aging and age-related diseases [243].

Epidemiological studies suggest that people with diabetes and taking metformin have lower incidences of age-related diseases and even exhibit a longer lifespan compared to that of non-diabetics [244]. These findings led to the TAME (Targeting Aging with Metformin) trial, a large-scale clinical study evaluating the effectiveness of metformin in delaying aging-related diseases [245]. If successful, this trial could pave the way for metformin to be repurposed as an anti-aging therapy.

**Table 1:** Metformin’s protective effects on different neuropathological conditions.

Pathological Factor	Triggering Factors	Metformin’s Protective Effects	Cited Reference
BBB Disruption	Proinflammatory cytokines and DAMPs	Inhibition of immune cell infiltration and inflammation	Chaves et al., 2024 [118]
Proinflammatory Cytokines	Mitochondrial dysfunction and apoptosis	Inhibition of neuroinflammation and protection from synaptic loss	Chung et al., 2015 [222]
Synaptic Dysfunction	Inflammation and oxidative stress	Inhibition of neuroinflammation and oxidative stress	Li et al., 2012 [246]
Neuronal Apoptosis	ROSs and mitochondrial dysfunction	Protection against neuronal loss	Chen et al. 2020 [247]
Mitochondrial Dysfunction	ROSs, cytokines, and DAMPs	Protection of mitochondrial function	Klemmensen et al., 2024 [223]
ROS Overproduction	Mitochondrial impairment and cytokines	Modulation of oxidative stress	Deng et al., 2023 [134]
DAMP Activation	Cell injury and protein aggregates	Inhibition of inflammasome/NF-κB activation	Zhang et al., 2010 [69]
NLRP3/NF-κB Activation	DAMPs and ROSs	Inhibition of cytokine release	Boaru et al., 2015 [248]

## 15. Conclusions

In conclusion, metformin can be a neuroprotective drug that decreases neuroinflammation and oxidative stress (Table 1). Metformin can be offered as a potential therapeutic alternative for neurodegenerative diseases, such as Alzheimer’s disease. Metformin reduces inflammation and oxidative damage while promoting the AMP-activated protein kinase (AMPK), which helps to maintain cellular energy homeostasis. Additionally, its anti-inflammatory properties decrease NF-κB activation, a primary source of neuroinflammation, while increasing Nrf2 activation, a crucial pathway for antioxidant defense. Furthermore, metformin promotes autophagy, which lessens neuronal damage by facilitating the elimination of cellular constituents and damaged protein aggregates. Regarding aging and cognitive function, metformin’s neuroprotective actions may enhance brain functions and reduce the risk of neurodegenerative diseases. Metformin protects neurons by preserving the integrity of the blood–brain barrier (BBB), thereby preventing toxic chemicals and inflammatory mediators from entering the brain. Furthermore, metformin modifies microglial activation, shifting the balance in favor of anti-inflammatory responses and reducing inflammation-induced damage. The evidence suggests that metformin’s neuroprotective properties, which are mediated via oxidative stress and the regulation of neuroinflammation, may hold great promise for preventing and treating neurodegenerative diseases.

## Data Availability

No new data were created or analyzed in this study.
